# Implementing PROMEHS to Foster Social and Emotional Learning, Resilience, and Mental Health: Evidence from Croatian Schools

**DOI:** 10.3390/children13010154

**Published:** 2026-01-22

**Authors:** Sanja Tatalović Vorkapić, Lidija Vujičić, Akvilina Čamber Tambolaš, Ilaria Grazzani, Valeria Cavioni, Carmel Cefai, Liberato Camilleri

**Affiliations:** 1 Faculty of Teacher Education, University of Rijeka, 51000 Rijeka, Croatia; lidija.vujicic@uniri.hr (L.V.); akvilinact@uniri.hr (A.Č.T.); 2 Department of Human Sciences for Education "R. Massa", University of Milano-Bicocca, 20126 Milano, Italy; ilaria.grazzani@unimib.it; 3 Department of Humanities and Social Sciences, Faculty of Society and Communication Sciences, Universitas Mercatorum, 00186 Rome, Italy; valeria.cavioni@unimercatorum.it; 4 Department of Psychology, University of Malta, MSD 2080 Msida, Malta; carmel.cefai@um.edu.mt; 5 Faculty of Science, University of Malta, MSD 2080 Msida, Malta; liberato.camilleri@um.edu.mt

**Keywords:** mental health, PROMEHS curriculum, resilience, SEL, students, teachers, well-being

## Abstract

**Background/Objectives**: In light of the concerning research data on students’ mental health, it is essential to provide high-quality programs that support children and young people in strengthening their psychological well-being. To address this need, the three-year Erasmus+ KA3 international project *PROMEHS: Promoting Mental Health at Schools* was developed. The project involved universities and education policy representatives from seven European countries, Italy (project leader), Greece, Croatia, Latvia, Malta, Portugal, and Romania. Its core activities included the development of the PROMEHS curriculum, grounded in three key components: social and emotional learning, resilience, and the prevention of behavioral problems, alongside a rigorous evaluation of its implementation. The main research aim was to test the effect of PROMEHS on students’ and teachers’ mental health. **Methods**: In Croatia, the curriculum was introduced following the training of teachers (N = 76). It was implemented in kindergartens, and primary and secondary schools (N = 32), involving a total of 790 children. Using a quasi-experimental design, data were collected at two measurement points in both experimental and control groups by teachers, parents, and students. **Results**: The findings revealed significant improvements in children’s social and emotional competencies and resilience, accompanied by reductions in behavioural difficulties. These effects were most evident in teachers’ assessments, compared to parents’ ratings and student self-reports. Furthermore, teachers reported a significantly higher level of psychological well-being following implementation. **Conclusions**: Bearing in mind some study limitations, it can be concluded that this study provides evidence of the positive effects of PROMEHS in Croatian educational settings. Building on these outcomes and PROMEHS as an evidence-based program, a micro-qualification education was created to ensure the sustainability and systematic integration of the PROMEHS curriculum into Croatian kindergartens and schools.

## 1. Introduction

With the advent of the 21st century, the everyday lives of children and young people have changed significantly [1,2]. Contemporary childhood and youth are shaped by a range of diverse and entirely new life circumstances which, under the influence of rapid technological advancement, continue to undergo profound transformation [3,4,5]. Collectively, these changes substantially influence the needs and interests of children and young people, as well as their overall quality of life, well-being, and mental health [6,7,8], both in general terms and more specifically within the context of upbringing and education [9]. Recent data indicate a continuously increasing prevalence of psychological difficulties among young people [5,10,11,12,13], implying a strong need for systematic support and proactive intervention through the development of high-quality, evidence-based programs aimed at strengthening their well-being and mental health [14]. This issue becomes even more pressing when the well-being and mental health of children and young people are examined within the educational context [15,16]. In the NESET report [17], produced by a network of experts specializing in social and emotional learning and educational dimensions of schooling, a clear consensus is articulated: the acquisition of purely academic (cognitive) knowledge, particularly when it is limited to factual and conceptual learning, as is often the case in schools, is insufficient for the holistic development of children and young people into active and responsible citizens. Such individuals should be capable of effectively and constructively addressing diverse life challenges, while achieving a balance between personal life satisfaction and ongoing personal and social development, both for themselves and for others within the communities in which they live and act. It is therefore evident that the contemporary educational needs of children and young people require a more complex and multi-layered response than mere academic knowledge and school achievement alone [18,19,20], yet these remain the predominant focus within many educational systems.

A meaningful response regarding what should be changed, and how it must include direct, explicit, and targeted concern for the mental health and well-being of all stakeholders involved in the educational process and system, is necessary. This necessity is further substantiated by alarming reports on the mental health of children and young people [21,22,23]. In addition to the previously noted increase in the number of children and adolescents experiencing various mental health difficulties, analyses also point to a significant decline in their levels of psychological well-being and life satisfaction [24,25]. According to the *Comprehensive Mental Health Action Plan 2013–2030* [26] over the past two decades, the rise in mental health problems among children and adolescents worldwide has become a leading cause of disability and school absenteeism. Suicide is the second most common cause of death among young people globally [26] (p. 2). The prevalence of diagnosed mental, emotional, or behavioral disorders among students is estimated to range between 10% and 20% [27]. The proportion of young people who have experienced mental health difficulties before the age of 14 and before the age of 18 is estimated at 35% and 48%, respectively, with the highest incidence occurring at approximately 14.5 years of age [28]. Depression and anxiety are among the five most common causes of illness, while suicide represents a leading cause of death among adolescents in countries with low and middle gross national income [29]. Furthermore, 35% of 13-year-old students and 40% of 15-year-olds in Europe reported feeling low in mood, nervous, or experiencing psychosomatic symptoms more than once per week [30]. In addition, 20% of adolescents aged 11 to 17 in Europe stated that they had grown up feeling unhappy and worried about their future as a result of experiences of abuse, academic pressure, and loneliness [31]. During the COVID-19 pandemic, a high proportion of individuals worldwide reported symptoms of anxiety, depression, and post-traumatic stress disorder [32,33], a finding also confirmed by national studies conducted among adults, children, and young people in Croatia [34,35,36]. All the above is further reflected in recent increases in violent forms of behaviour within Croatian schools [37] (p. 45).

### 1.1. Schools as Ecosystems That Support Children’s Mental Health and Well-Being

The discourse on the unjust marginalization of the nurturing role of schools has been emphasized for more than two decades [18]. The continuous enhancement of the educational and developmental role of schools is of critical importance, as is the integration of life-essential and indispensable student competencies that are closely aligned with those of teachers [38,39]. On the one hand, research clearly indicates that a high-quality educational continuum from early childhood onwards should exert an equally positive influence on all domains of child development, rather than focusing exclusively on cognitive outcomes [19,40,41]. On the other hand, the circumstances of contemporary life impose a range of challenges that require adequate responses, particularly through a focused strengthening of children’s and young people’s mental health and well-being within the environments in which they spend most of their time, namely, within schools [42,43]. This does not imply that schools should become psychotherapeutic institutions; rather, it suggests that educational systems should be designed in ways that bring learning processes closer to natural, intrinsically motivated, and self-regulated learning. Within such frameworks, students are provided with opportunities to develop self-knowledge and an understanding of others, recognize their own and others’ potentials and strengths, and cultivate not only cognitive but also social and emotional competencies. Furthermore, this approach supports the development of a positive self-concept and self-esteem, the acquisition of essential life skills, empathy towards others, and tolerance of diversity in all its forms. The effectiveness of such holistic frameworks is well-documented in international literature; for instance, large-scale meta-analyses [41,44,45] demonstrate that systemic SEL interventions significantly improve students’ social–emotional skills and academic performance across diverse cultural contexts. By adopting this ecosystemic perspective, schools transition from purely instructional sites to ‘health-promoting’ environments that foster long-term resilience [16].

A specific strategic objective identified by the World Health Organization [26] in the field of global mental health support explicitly recognizes the pivotal role of educational systems in addressing the mental health needs of children and young people, as well as in enhancing their quality of life and psychological well-being. The World Health Organization recommends that schools are functioning as one of the core mental health support systems for students, enabling promotion, prevention, and intervention. Approximately half of all mental health problems emerge before the age of 14, when students are still attending school, which creates a significant opportunity for the active promotion of mental health and well-being within school settings, as well as for the early identification of difficulties and timely prevention and intervention during critical developmental periods. Children’s and young people’s mental health is not an isolated phenomenon but rather an integral component of the dynamic interaction between individuals and their environments. Consequently, it is essential to develop school practices and policies that systematically integrate mental health promotion into the educational environment, as recommended by the *School Mental Health Theoretical Framework* [46,47]. This theoretical model is grounded in a whole-school approach [48,49], which foregrounds multilevel intervention strategies aimed at embedding mental health promotion across all aspects of school functioning through collaborative engagement of students, teachers, parents, and school leaders.

A fundamental principle of this approach is that schools cannot effectively promote students’ mental health if the mental health and well-being of teachers themselves are not addressed [48], a notion aptly captured in the well-known statement *“Well teachers, well students”* [50] (p. 19). Accordingly, it is insufficient to merely equip teachers with the competencies required to foster students’ well-being and mental health; rather, it is crucial to ensure a strong sense of teachers’ professional well-being and psychological health [38,39,51]. This emphasis is central to the *School Mental Health Theoretical Framework* [47], which encompasses three core components: social and emotional learning (SEL), resilience, and the prevention of social, emotional, and behavioral difficulties. These components also constitute the foundational basis for the development of the PROMEHS curriculum.

### 1.2. Creating, Implementing, and Testing the PROMEHS Curriculum in Croatia

To ensure the effective promotion of mental health and well-being within educational practice in schools, a fundamental prerequisite is the high-quality development of teachers’ professional competencies, alongside their own positive mental health and high levels of well-being. National studies [39,52,53] and international analyses [47,54,55] have consistently demonstrated a very strong need among teachers for strengthening their competencies in the field of mental health and well-being. Despite the established value of SEL, a significant research gap remains regarding the implementation of such curricula in Southeast European countries like Croatia, which face unique systemic challenges and rapid social transitions. Most existing large-scale studies have been conducted in Western, high-income contexts, leaving a void in evidence-based knowledge on how such programs function in more resource-constrained educational systems. This study addresses this gap by providing a methodologically robust evaluation of the PROMEHS curriculum’s impact on both students and teachers within the specific socio-cultural landscape of Croatian schools, thereby advancing the international understanding of SEL scalability and cultural adaptation.

In response to these needs and to address this evidence gap, within the Erasmus+ KA3 international project *PROMEHS: Promoting Mental Health at Schools* [56], implemented between 2019 and 2022, the PROMEHS curriculum was developed, implemented, and evaluated for students aged 3 to 18 years. The project involved seven European countries: Greece, Croatia, Latvia, Malta, Portugal, Romania, and Italy, with Italy serving as the project leader and main coordinator. From Croatia, participants included researchers from the Faculty of Teacher Education in Rijeka, as well as representatives of educational policy from the Department of Education of the City of Rijeka and the Administrative Department for Education of the Primorje and Gorski Kotar County.

The primary objective of the project was to develop, through collaboration among university representatives, educational policy makers (ministries), scientific associations, and educational practitioners, a curriculum grounded in the *School Mental Health Theoretical Framework* [46]. This framework also constitutes the central theoretical foundation of the present study. It encompasses the social and emotional learning (SEL) process, within which individuals acquire and apply knowledge, skills, and attitudes to develop healthy identities, manage emotions, achieve personal and collective goals, demonstrate empathy, establish supportive relationships, and make responsible decisions [57,58]. SEL represents a core component of teacher well-being, as teachers assume a dual role in managing their own emotional health while simultaneously fostering these competencies in their students. SEL comprises five key dimensions: self-awareness, self-management, social awareness, relationship skills, and responsible decision making. A substantial body of evidence points to numerous positive outcomes associated with SEL implementation, including more effective classroom management, improved teacher-student relationships, a more positive school climate, enhanced coping in challenging situations involving students with behavioral difficulties, and more constructive collaboration with colleagues and parents [41,59,60,61]. In addition, the theoretical framework incorporates resilience, defined as the capacity to adapt effectively to adversity and to recover rapidly from life challenges [62]. Higher levels of resilience are associated with a greater ability to maintain well-being at an optimal and personally appropriate level. In the present study, resilience encompassed two key domains: dealing with psychosocial challenges and dealing with traumatic experiences. The third component of the framework addresses the prevention of social, emotional, and behavioural difficulties, including dealing with internalizing problems, dealing with externalizing problems, and dealing with at-risk behaviors [63]. Beyond the core theoretical model and the whole-school approach, the development of the new mental health curriculum was also guided by several key principles: inclusiveness, interculturality, sustainability, simplicity, a holistic perspective, methodological and didactic quality, and strong scientific and practical grounding of developmentally sensitive handbooks tailored to students aged 3 to 18 years. Following the curriculum development phase, structured training was delivered in all participating countries to teachers involved in curriculum implementation within their schools. This enabled the subsequent evaluation phase of the project to assess the effectiveness of the PROMEHS curriculum.

## 2. The Current Study

The research methodology was standardized across all countries participating in the implementation, as reflected in the cross-national research findings [64,65]. The specific aim of the present study was to examine the PROMEHS effect within the Croatian sample. From this general research aim, the following research questions were derived:

(a) To examine the effect of the implemented PROMEHS curriculum on students’ mental health based on three types of assessments: teacher reports, parent reports, and student self-reports; and
(b) To examine the effect of the implemented PROMEHS curriculum on teachers’ mental health based on their self-reports.

For both research questions, a positive effect of the implemented PROMEHS curriculum on the mental health of students and teachers was expected.

## 3. Materials and Methods

### 3.1. Participants

A random sample was drawn based on the expressed interest of kindergartens and schools in Primorje and Gorski Kotar County. Following the initial expression of interest, a geographically proximate cluster sample of institutions located near the Faculty of Teacher Education agreed to participate in the study. The research materials included an invitation letter addressed to school principals; informed consent forms for teachers, parents, and students; two ethical approvals (one issued by the Ministry of Science and Education and one by the University of Rijeka); and a set of relevant questionnaires. These materials were distributed to a total of 1023 students and parents, as well as 88 teachers, encompassing 15 kindergartens, 18 primary schools, and 7 secondary schools. Subsequently, informed consent for participation was obtained from a total sample of N = 790 children (with student age categories assessed by teachers), N = 76 teachers (aged 18–29 years: 9 teachers; 30–39 years: 36 teachers; 40–49 years: 20 teachers; 50–59 years: 10 teachers; 60 years and older: 1 teacher), and N = 453 parents (data on parents’ age and gender were not collected). These participants were recruited from 14 kindergartens, 14 primary schools, and 4 secondary schools. The mean age of students who completed self-report measures was M = 16.28 years (SD = 0.93), with ages ranging from 12 to 17 years. Socio-demographic characteristics of the sample are presented in Table 1.

### 3.2. Measures

Different measures were employed based on three criteria: (a) the target of the assessment (students or teachers), (b) the type of rater (teachers, students, or parents), and (c) the type of rating (other-report or self-report). In this section, the measures are presented according to the first criterion, distinguishing between instruments used to assess students (as rated by teachers, parents, and the students themselves) and instruments used to assess teachers (teachers’ self-reports). All instruments underwent a back-translation procedure conducted by Croatian researchers involved in the project, as well as by an independent Croatian expert in the English language. The only exception was the Strengths and Difficulties Questionnaire (SDQ), which had previously been translated into Croatian by Helena Hamilton and Nataša Momčilović and was therefore used in its existing validated Croatian version [66].

#### 3.2.1. Student Measures

A set of three masures was applied for measuring the effect of PROMEHS on students: a Strengths and Difficulties Questionnaire [67] for measuring internalized and externalized difficulties and strength (prosocial behavior); SSIS-SEL [68] for measuring social and emotional competencies—self-awareness, self-management, social awareness, relationships, and responsible decision making; and CD10: the Connor–Davidson resilience Scale [69] for measuring resilience. Teachers and parents used the first two measures for rating students, and students used all three measures for self-rating. These tools show the best psychometric properties and are widely used in research to measure difficulties, behaviors, resilience, and social and emotional skills [65].

*The Strengths and Difficulties Questionnaire* (SDQ; [69]) is a brief screening instrument designed to assess the mental health of students aged 3 to 16 years. It can be completed by teachers, parents, and students aged 11 years and older. The questionnaire comprises 25 items organized into five subscales: conduct problems and hyperactivity (together forming the *Externalizing Problems* dimension), emotional symptoms and peer relationship problems (together forming the *Internalizing Problems* dimension), and a *Prosocial Behavior* subscale. The first four subscales jointly reflect students’ difficulties. In the present study, the three-factor model consisting of Internalizing Problems, Externalizing Problems, and Prosocial Behavior was applied [70]. All items were rated on a 3-point Likert scale. In the original validation study, the subscales demonstrated acceptable internal consistency, with Cronbach’s alpha coefficients of 0.66, 0.76, and 0.66, respectively [70]. In this current study, the Cronbach’s alpha of the ten items measuring the internalizing problem were 0.719 (teacher evaluations), 0.793 (parent evaluations), and 0.728 (student self-evaluations). The Cronbach’s alpha of the ten items measuring the externalizing problem were 0.693 (teacher evaluations), 0.702 (parent evaluations), and 0.690 (student self-evaluations). The Cronbach’s alpha of the five items measuring prosocial behavior were 0.771 (teacher evaluations), 0.782 (parent evaluations), and 0.686 (student self-evaluations). Moreover, for the teachers’, parents’, and students’ rating evaluations, the three-factor model was confirmed by confirmatory factor analysis, which yielded satisfactory fit-indices.

*The Social Skills Improvement System, Social Emotional Learning Edition Brief Scales—Student Form* (SSIS-SELb-S; [68,71,72,73]) is a measure of social and emotional competence in children and adolescents aged 3 to 18 years and can be completed by teachers, parents, and students. Based on the Collaborative for Academic, Social, and Emotional Learning [73] framework, the instrument assesses five core domains: self-awareness, self-management, social awareness, relationship skills, and responsible decision making. The scale consists of 20 items, with each of the five subscales comprising four items rated on a 4-point Likert scale. SSIS-SELb-S has demonstrated high reliability, with a Cronbach’s alpha of 0.91 for the composite score and coefficients ranging from 0.67 to 0.72 for the five subscales [74]. In this current study, the Cronbach’s alpha of the four items measuring self-awareness were 0.791 (teacher evaluations), 0.727 (parent evaluations), and 0.631 (student self-evaluations). The Cronbach’s alpha of the four items measuring self-management were 0.789 (teacher evaluations), 0.716 (parent evaluations), and 0.682 (student self-evaluations). The Cronbach’s alpha of the four items measuring social awareness were 0.872 (teacher evaluations), 0.816 (parent evaluations), and 0.719 (student self-evaluations). The Cronbach’s alpha of the four items measuring relationship skills were 0.711 (teacher evaluations), 0.729 (parent evaluations), and 0.619 (student self-evaluations). The Cronbach’s alpha of the four items measuring responsible decision making were 0.813 (teacher evaluations), 0.827 (parent evaluations), and 0.676 (student self-evaluations). Moreover, for the teachers’, parents’, and students’ rating evaluations, the five-factor model was confirmed by confirmatory factor analysis, ensuring that the theoretical construct is well-represented.

*The Connor–Davidson Resilience Scale* (CD-RISC-10; [75]) is a self-report measure assessing individuals’ capacity to cope with adversity. The short version consists of 10 items rated on a 5-point Likert scale ranging from 0 to 4 [69]. The instrument is primarily intended for students aged 13 to 17 years, although it can also be administered to students as young as 10 years. In the original validation study, the scale demonstrated good internal consistency, with a Cronbach’s alpha of 0.85. In this current study, the Cronbach’s alpha of the ten items measuring student’s self-reported resilience was 0.784.

#### 3.2.2. Measures for Teachers’ Self-Ratings

Four measures were used for testing the effect of PROMEHS on teachers’ mental health and well-being. Teacher Sense of Efficacy—Short Version [76] was applied for measuring self-efficacy dimensions of student engagement, instructional strategies, and classroom management. The Connor–Davidson Resilience Scale—CD 10 [77] was used for measuring teachers’ resilience. The Social and Emotional Competence of Teachers Scale (SECTRS) [77] was used to measure teachers’ social and emotional competencies across the following dimensions: teacher-student relationships, emotional regulation, social awareness, and interpersonal relationships. In addition, teachers’ burnout was assessed using simple as exploratory variable with a single item: *“I feel exhausted at the end of the working day”*, developed by members of the PROMEHS project.

*The Ohio State Teacher Efficacy Scale* (OSTES; [76]) is a self-report questionnaire designed to assess teachers’ sense of efficacy across three dimensions: *student engagement*, *instructional strategies*, and *classroom management*. Each dimension is measured using four items, yielding a total of 12 items, which are rated on a 9-point Likert scale ranging from 1 (*nothing*) to 9 (*a great deal*). In the original validation study, the OSTES demonstrated satisfactory levels of internal consistency, with Cronbach’s alpha coefficients ranging from 0.81 to 0.86 [76]. In this current study, the Cronbach’s alpha of the four items measuring student engagement was 0.831; the Cronbach’s alpha of the four items measuring instructional strategies was 0.701; and the Cronbach’s alpha of the four items measuring classroom management was 0.770. Moreover, confirmatory analysis confirmed the three-factor model yielding satisfactory fit-indices.

*The Connor–Davidson Resilience Scale* (CD-RISC-10; [75]) consists of 10 items and assesses teachers’ capacity to cope with adversity. Responses are provided using a 5-point Likert scale. The original validation study reported satisfactory internal consistency (α = 0.85). In this current study, the Cronbach’s alpha of the ten items measuring teachers’ self-reported resilience was 0.826.

*The Social and Emotional Competence in Teachers Scale* (SECTRS; [77]) comprises 52 items and measures four domains of social and emotional competence in accordance with the CASEL framework [73]. Teachers provide self-ratings on a 6-point Likert scale ranging from 1 (*strongly disagree*) to 6 (*strongly agree*). Scores reflect teachers’ perceived levels of competence across the following domains: *teacher–student relationship* (7 items), *emotional regulation* (6 items), *social awareness* (6 items), and *interpersonal relationships* (6 items). The original study reported satisfactory levels of internal consistency, with Cronbach’s alpha coefficients ranging from 0.69 to 0.81 [77]. In this current study, the Cronbach’s alpha of the seven items measuring teacher-student relationships was 0.807; the Cronbach’s alpha of the six items measuring emotional regulation was 0.789; the Cronbach’s alpha of the six items measuring social awareness was 0.813; and the Cronbach’s alpha of the six items measuring interpersonal relationships was 0.735. The four-factor model was confirmed using confirmatory factory analysis.

### 3.3. Research Design and Procedure

After the methodological procedures had been harmonized across all PROMEHS project partner countries, the first step in the administrative procedure of the project was obtaining ethical approval from two ethics committees: the Faculty of Teacher Education, University of Rijeka, and the Ministry of Science and Education of the Republic of Croatia. The administrative procedure was organized across twelve phases or steps. Participant recruitment was performed in collaboration with the founders of kindergartens, primary schools, and secondary schools in Primorje and Gorski Kotar county, i.e., the Department of Education of the City of Rijeka and the Administrative Department for Education of the Primorje and Gorski Kotar County, who were project partners. Their representatives provided the telephone and e-mail contacts of all kindergartens, primary schools, and secondary schools in the county. Regarding the inclusion and exclusion criteria, all interested schools are included in the study; and private founded schools were not included due to possible differences in their organization. So, the second step in the administrative procedure was contacting all schools in the county in collaboration with administrative education offices in the City of Rijeka and the Primorje and Gorski Kotar County. In July 2020, an initial call for expressions of interest to participate in the study was disseminated to all kindergartens, primary schools, and secondary schools in Primorje and Gorski Kotar County. A total of 40 educational institutions expressed interest in collaborating on the project. The main objectives and research timeline were subsequently presented to representatives of all kindergartens and schools in the county at the National PROMEHS Opening Conference held on 14 October 2020, which was the third step in the administrative procedure. Follow-up meetings with school principals as the forth administrative step took place on 5 and 6 November 2020. A set of prepared questionnaires in the sixth step of the administrative procedure was then distributed to 1023 children and parents, as well as 88 teachers, across 15 kindergartens, 18 primary schools, and 7 secondary schools. Based on agreements reached during meetings with school principals, the final participating sample comprised N = 32 educational institutions, including 14 kindergartens, 14 primary schools, and 4 secondary schools. Informed consent was obtained from all participants in the final sample, including students (N = 790), teachers (N = 76), and parents (N = 453). All participating kindergartens and schools were randomly assigned to either the experimental group or the control (waiting list) group. A training course for the experimental group was conducted on 27 and 28 November 2020 as the seventh step in the administrative procedure. The training was accredited by the Education and Teacher Training Agency and included the provision of printed PROMEHS handbooks. It was delivered by members of the Croatian project team and external experts in the fields of social and emotional learning and teacher mental health. Following the training, pre-test data collection was carried out for both the experimental and control groups between November 2020 and February 2021.

All participants completed three online questionnaires via the SurveyMonkey platform (teachers assessed both students and themselves; parents assessed their children, and students completed self-assessments). To ensure participants’ privacy and to facilitate the matching of pre- and post-test data, teachers were assigned unique anonymized codes to be used when completing the questionnaires. The entire data collection process was conducted in coordination with and under the supervision of school principals, ensuring both anonymity and confidentiality of the collected data. Additionally, all data collected across the participating countries, including Croatia, were consolidated into a single database at the University of Malta. The University of Malta was not involved in data collection but was responsible solely for data processing, thereby ensuring a high level of objectivity and impartiality in the research results.

After the pre-test phase in both groups, the PROMEHS program was implemented only in the experimental group from 18 January to 15 June 2021, as the eighth step of the administrative procedure. During the implementation, teachers were encouraged to select activities based on two levels of complexity, basic or advanced, and to carry them out over a minimum period of 12 weeks. According to the previously described PROMEHS curriculum for mental health, each of the three units—social and emotional learning (SEL), resilience, and risk behaviors—comprised a set of topics with clearly defined goals and methods for the development of specific competencies. Skills were taught through structured activities appropriate to the students’ age. Each PROMEHS session lasted between 30 min and two school hours. Teachers were trained to effectively use the PROMEHS handbooks to successfully conduct the activities. The activities included stories, games, motor exercises, songs, online resources, and other interactive materials. Each activity provided a brief description of learning outcomes, target age, level of complexity, and the necessary materials for implementation. Activities followed a structured sequence: a short story featuring one or more main characters of the PROMEHS program, self-reflection questions to deepen discussion, practical exercises using various methodologies, and a qualitative evaluation chart for monitoring student progress. Methodological guidance for teachers included explanations of activity objectives, detailed instructions for integrating target skills into everyday practice, and links to media content and additional literature. The PROMEHS implementation also included monthly teacher supervision sessions, organized and led by the Croatian project coordinator in the ninth step of the administrative procedure. Simultaneously, webinars were conducted for parents participating in the study, first targeting parents of the experimental group and later those of the control group. Post-test data collection was carried out in May–June 2021 for both groups as the tenth step of the administrative procedure. Following this, closing meetings were held with schools from both the experimental and control groups. In addition, a training course for the control group was organized on 24 and 25 June 2021 as the eleventh step of the administrative procedure. Participants received free training, printed PROMEHS materials, and certificates from the Education and Teacher Training Agency. Within the final, twelfth step of the administrative procedure, the Closing National PROMEHS Conference was held on 28 January 2022, and included presentations of preliminary research results, lectures on students’ and teachers’ well-being and mental health, and roundtable discussions.

### 3.4. Data Analysis

For data analysis, students were matched using their assigned codes to combine pre- and post-test results, including only those students who had completed both assessments. Missing values were sparse and imputation was carried out by replacing the missing value with the mean score for the respective scales. The Kolmogorov–Smirnov test was used to examine the distribution of scores for each subscale. The distributions of internalizing and externalizing problems were positively skewed, whereas the distributions of prosocial behavior, and social and emotional learning, were negatively skewed and did not meet the assumption of normality. Therefore, non-parametric tests were employed to examine significant differences between the experimental and control groups, as well as within these groups before and after the PROMEHS implementation. The Mann–Whitney test is used to compare mean rating scores between two groups of participants (control vs. experimental) when the score distribution violates the normality assumption. The Wilcoxon test is used to compare mean rating scores between two time phases (phase 1 and phase 2) when the score distribution violates the normality assumption. The null hypothesis specifies that the mean rating scores vary marginally between the two groups/phases and is accepted if the *p*-value exceeds the 0.05 level of significance. The alternative hypothesis specifies that the mean rating scores vary significantly between the two groups/phases, and is accepted if the *p*-value is less than the 0.05 criterion.

## 4. Results

Given the primary aim of the study, which was to evaluate the effects of the PROMEHS program on the Croatian sample of students and teachers, the results are presented in accordance with the predefined specific research tasks.

### 4.1. PROMEHS Effects on Students Based on Teachers’ and Parents’ Ratings and Self-Ratings

Table 2, Table 3 and Table 4 present means and standard deviations to measure central tendency and dispersion. The Mann–Whitney test was used to compare mean scores between the control and experimental groups, while the Wilcoxon signed-rank test was used to compare mean scores between pre-test and post-test. For each test, the z-score, *p*-value, and Cohen’s d are provided to assess statistical significance and effect size. Moreover, the student variables considered include internalized and externalized difficulties, prosocial behavior, self-awareness, self-management, social awareness, relationships, responsible decision-making, total social and emotional competence (SEC), and students’ resilience based on self-ratings.

As shown in Table 2, according to teachers’ assessments, mean scores at pre-test of the control group were significantly higher than the experimental group in prosocial behavior (Z = −3.092, *p* = 0.002, and d = 0.462) and social awareness (Z = −3.163, *p* = 0.002, and d = 0.613). Mean scores at post-test for the control group were significantly higher than the experimental groups in social awareness (Z = −2.142, *p* = 0.032, and d = 0.615). Analyses within each group showed no statistically significant differences between pre- and post-test measurements in the control group, as expected. In contrast, according to teachers’ assessments, the experimental group exhibited significant differences in all variables except for self-management: students’ internalized and externalized difficulties (Z = −3.008, *p* = 0.003, and d = 0.292), prosocial behavior (Z = −2.420, *p* = 0.016, and d = 0.474), self-awareness (Z = −2.891, *p* = 0.004, and d = 0.563), social awareness (Z = −2.110, *p* = 0.035, and d = 0.620), relationships (Z = −2.059, *p* = 0.039, and d = 0.591), responsible decision-making (Z = −2.779, *p* = 0.005, and d = 0.617), and total social and emotional competencies (Z = −2.570, *p* = 0.010, and d = 0.525). All these variables increased significantly after PROMEHS implementation, except for internalized and externalized difficulties, which decreased significantly.

Table 3 presents parents’ assessments of students in both groups and across both testing phases. No significant differences were observed between control and experimental groups at pre-test. Mean scores at post-test of the control group were significantly higher than the experimental group in internalized and externalized difficulties (Z = −3.723, *p* < 0.001, and d = 0.216). Mean scores at post-test of the experimental group were significantly higher than the control group in responsible decision-making (Z = −2.121, *p* = 0.034, and d = 0.477), and total SEC (Z = −2.025, *p* = 0.043, and d = 0.361). Within-group analyses showed that in the control group, parents reported significantly higher post-test scores in self-management (Z = −2.272, *p* = 0.023, and d = 0.502). In the experimental group, parents reported significantly higher post-test scores for self-awareness (Z = −1.946, *p* = 0.050, and d = 0.435) and total social and emotional competencies (Z = −2.114, *p* = 0.035, and d = 0.365), and significantly lower post-test scores for internalized and externalized difficulties (Z = −2.922, *p* = 0.003, and d = 0.194).

According to students’ self-ratings (Table 4), mean pre-test scores were significanty higher for the experimental group than the control group in prosocial behavior (Z = −2.161, *p* = 0.031, and d = 0.362), which was higher in the experimental group. For each measure, the mean scores at post-test did not vary significantly between control and experimental groups. Within-group analyses showed that mean post-test scores of students in the control group were significantly higher in self-awareness (Z = −2.001, *p* = 0.045, and d = 0.443). The mean scores of students in the experimental group who participated in PROMEHS implementation were significantly higher in responsible decision-making (Z = −2.103, *p* = 0.035, and d = 0.391) and total SEC (Z = −1.957, *p* = 0.050, and d = 0.340).

### 4.2. PROMEHS Effects on Teachers Based on Their Self-Ratings

Table 5 presents the results of the Mann–Whitney test to compare mean scores between the control and experimental groups; and the Wilcoxon signed-rank test to compare mean scores between pre- and post-test for teachers’ self-rated variables. These include social and emotional competencies, resilience, self-efficacy (student engagement, instructional strategies, and classroom management), and burnout. Moreover, Cohen’s d was used to measure effect size. No statistically significant differences were observed between the control and experimental groups at pre-test. Mean scores at post-test of the experimental group were significantly higher than the control group for social and emotional competencies (Z = −2.207, *p* = 0.027, and d = 0.348) and all dimensions of self-efficacy: student engagement (Z = −2.553, *p* = 0.011, and d = 0.834), instructional strategies (Z = −2.436, *p* = 0.015, and d = 0.721), and classroom management (Z = −3.410, *p* = 0.001, and d = 0.714), with all these mental health and wellbeing indicators being significantly higher in the experimental group. Within-group analyses revealed no significant changes in the control group between pre-test and post-test. In contrast, as expected, the experimental group showed significant increases from pre-test to post-test in teachers’ resilience (Z = −2.447, *p* = 0.014, and d = 0.433), and all self-efficacy dimensions: student engagement (Z = −2.041, *p* = 0.041, and d = 0.872), instructional strategies (Z = −2.425, *p* = 0.015, and d = 0.835), and classroom management (Z = −2.032, *p* = 0.042, and d = 0.814).

## 5. Discussion

### 5.1. PROMEHS Effectiveness in Students’ Sample from Three Perspectives

Students’ internalized and externalized behavior difficulties, prosocial behavior, five dimensions of social and emotional competencies, and total social and emotional competencies were assessed pre- and post-implementation of the PROMEHS program in the experimental group. To evaluate the effectiveness of the PROMEHS curriculum on students’ well-being and mental health, operationalized through the aforementioned variables, a quasi-experimental design was applied. Significant differences were expected between pre- and post-test in the experimental group, as well as between experimental and control groups at post-test. Overall, most findings from multiple perspectives confirmed the formulated hypotheses, although some non-significant results should be interpreted with caution.

The effectiveness of PROMEHS on students’ mental health was evaluated from three perspectives: teachers, parents, and students themselves, using the same measures (with the addition of a resilience self-rating for students). According to teachers’ assessments, a significant positive effect of the PROMEHS curriculum on students’ mental health was observed. This effect was evident across all domains of social and emotional learning, internalized and externalized behavior problems, and prosocial behavior. Even though the significance of differences was less pronounced in parents’ and students’ assessments, according to determined z-values and effect sizes, their ratings are complementary with teachers’ ratings, bearing in mind different context-bound perspectives from multi-informant findings. Regarding context-depending ratings in various raters, it is expected to find differences due to their various perspectives [78]. Based on teachers’ ratings, the most consistent and stable significant differences were found within the experimental group between pre- and post-test. In majority of tested differences, moderate effect sizes were determined. No significant changes were observed in the same variables within the control group, as expected. These findings suggest that, following PROMEHS implementation, students’ internalized and externalized behavior problems decreased, while prosocial behavior, self-awareness, social awareness, relationships with others, responsible decision-making, and overall social and emotional competencies increased. Although self-management showed a positive trend, the difference was not statistically significant.

These results are consistent with findings from other PROMEHS project member countries [64,65,72,79]. However, the significant differences observed within the experimental group were not consistently accompanied by the expected significant differences between experimental and control groups at post-test, except for the variable “relationships with others.” Therefore, the observed improvements in mental health cannot be entirely attributed to the PROMEHS intervention, as other factors, including developmental effects, may have contributed. This outcome may be explained by the fact that the control group had higher baseline scores on most variables compared to the experimental group, with prosocial behavior and social awareness showing significant pre-test differences. The research groups were therefore not fully equivalent in terms of students’ well-being and mental health at baseline. This inequality may have arisen because only separate experimental and control schools were used to avoid contact between the groups within the same school. Consequently, group assignment may have affected the balance of baseline characteristics, which might have been more comparable if both groups had been drawn from the same schools and shared the same school climate. Future studies should consider creating experimental and control groups within the same schools to improve baseline equivalence.

In order to discuss the findings on students’ mental health from the perspective of their parents, a different pattern of differences was observed. Prior to further discussion, one should have in mind that parents ar emono heterogeneous sample than teachers and they had no previous education (as teachers did) on the variables that were rate din students. Also, discrepancies between teachers’ and parents’ ratings were expected due to different context-bound perspectives [80], the results of this study revealed a twofold pattern. First, a smaller number of significant differences were found within the experimental group between pre- and post-test, accompanied with low to moderate effect sizes, indicating a relatively small PROMEHS effect compared to that observed in teachers’ ratings. Specifically, after PROMEHS implementation, students in the experimental group exhibited significantly higher levels of prosocial behavior and overall social and emotional competencies, as well as significantly lower levels of internalized and externalized behavior problems. On the other hand, parents’ ratings revealed significant differences between groups at post-test, suggesting that some observed changes could be fully attributed to the PROMEHS intervention. In particular, this applied to the reduction in students’ internalized and externalized behavior problems and the increase in overall social and emotional competencies. Convergence between teachers’ and parents’ ratings was observed in students’ self-management scores, where no significant differences in the expected direction were detected. Moreover, parents’ ratings indicated that self-management was significantly higher in the control group between pre- and post-test. Although this finding may be influenced by the unplanned effects of the COVID-19 pandemic, which coincided with the start of PROMEHS implementation, it represents an interesting result that warrants further investigation in future studies.

Finally, considering students’ self-ratings, PROMEHS effects were perceived in only two domains: responsible decision-making and overall social and emotional competencies, with low to moderate effect sizes. However, these differences in the experimental group were not accompanied by significant between-group differences at post-test, and therefore cannot be entirely attributed to the PROMEHS intervention. Additionally, the groups were not equivalent in terms of students’ self-assessment of prosocial behavior, and a significant increase in self-awareness was observed in the control group between pre- and post-test. In interpreting these results, it is important to consider different perspectives of students and their understanding of the scales, and the relatively small number of students who completed the self-ratings. Future studies should address these issues by providing more detailed instructions for completing the scales [81] and by involving students in the design of PROMEHS activities, rather than limiting their role to program evaluation.

### 5.2. PROMEHS Effectiveness in Teachers’ Sample

The most consistent and stable findings with the highest effect sizes in this study, regarding the effectiveness of PROMEHS, were observed in teachers’ mental health. These results not only align with prior evaluations of the PROMEHS curriculum [53,64,65], but also substantiate broader international research demonstrating the positive impact of social and emotional learning interventions on teacher well-being, stress reduction, and self-efficacy [82,83,84]. No statistically significant differences were found between groups across the research phases or within the control group between pre- and post-testing. In contrast, significant differences were observed as expected in the experimental group, both between pre- and post-testing and between groups at post-test. Specifically, teachers reported significantly higher levels of resilience and all three dimensions of professional self-efficacy: student engagement, instructional strategies, and classroom management, after PROMEHS implementation. These differences are likely attributable to the PROMEHS program, as confirmed by significant between-group differences at post-test, with teachers in the experimental group exhibiting higher self-efficacy scores.

Although the findings regarding teachers’ social and emotional competencies and resilience are less conclusive, the relatively small sample size (N = 76) should be taken into account. Additionally, while a reduction in teachers’ burnout was observed in the experimental group, no statistically significant differences were detected, possibly because only a single item was used to measure burnout. Caution should be held on this exploratory variable due to the single item used. This should be reiterated to the value of validated multi-item burnout measures in future work, so future studies could be able to employ a validated and reliable burnout scale with a larger teacher sample. Nevertheless, the overall impact of providing psychological support to teachers during PROMEHS implementation was highly positive, as evidenced by qualitative data [64,65]. Teachers reported that the PROMEHS handbooks were extremely valuable resources, offering comprehensive methodological and didactic preparation for activities, which facilitated both in-person and online implementation. Supervision sessions, conducted online to share experiences and support joint learning, were also positively evaluated. Teachers indicated that PROMEHS activities fostered more open and effective communication with parents and observed positive changes in student behavior, particularly among those who exhibited behavioral difficulties prior to implementation. Finally, the Croatian project team received very positive feedback, both directly from teachers and indirectly from participating parents, who found the PROMEHS program engaging, useful, and beneficial for their children.

## 6. Conclusions

Although this study demonstrated several methodological limitations, as previously described, the PROMEHS curriculum for mental health showed at certain points to be an effective universal mental health program for early childhood, primary, and secondary schools in Europe, particularly in enhancing students’ and teachers’ mental health and well-being. Significant improvements were observed mostly based on teachers’ ratings with mostly moderate size effects. Additionally, the parents ratings and students’ self-ratings were not so supportive regarding PROMEHS implementation in the experimental groups. Finally, the positive effects, especially regarding the effect sizes, were mostly pronounced in the sample of participating teachers in this study, regarding their self-ratings. Since a very recent systematic review of cross-national research on adolescent mental health [85] has clearly demonstrated that there is a significant lack of validated measures across countries for measuring different aspects of adolescent mental health with an addition that internalizing problems and bullying are well-researched, whereas externalizing behaviors receive less attention, the contribution of this study is twofold: in validating new mesures and exploring externalized behaviours as well.

To address the already discussed study limitations, several recommendations for future studies emerge from this research: (a) to create experimental and control groups within the same schools rather than in different schools, ensuring equivalent baseline levels of well-being and mental health; (b) to use random sampling and increase the number of participants (in this study, the number of rated students was N = 790, parents N = 453, and students who completed self-ratings N = 200); and (c) to employ reliable and valid scales with detailed instructions, especially for parents and students. There is a strong need for longitudinal, systematic, and continuous implementation of the program, including integration into curricula, application of the SAFE approach, and quality implementation through education, mentoring, and monitoring. The fundamental principle of the PROMEHS curriculum is a holistic, whole-school approach, that encompasses the development of competencies across three domains for all stakeholders in the educational system: students, teachers, parents, and educational policymakers. Additionally, there is an emphasis on the contemporary role of teachers, which requires continuous and systematic improvement, supported by higher education institutions through initial training, lifelong education, and professional development programs.

In line with PROMEHS sustainability, a new educational program at the Faculty of Teacher Education, University of Rijeka, Croatia, titled *Mental Health and Well-Being in Education*, was launched in the academic year 2022/2023. This micro-qualification program operates in two modalities: as part of two study programs (Teaching Studies and Early and Preschool Care and Education), and as a lifelong learning program for kindergarten and primary school teachers. The program was developed within the UNIRI_CLASS A1 infrastructure project: *Personalized Education*, which lasted one year and was funded by the University of Rijeka. The program represents a continuation of PROMEHS curriculum implementation in kindergartens and primary schools. Twelve professors, along with guest lecturers, deliver the program with the aim of achieving defined learning outcomes. Participants also receive free PROMEHS handbooks to support ongoing work in kindergartens and primary schools. The program comprises three courses totaling 12 ECTS credits: *Mental Health, Well-Being, and Professional Development of Teachers*; *Mental Health and Well-Being of Children in the Educational Context*; and the *PROMEHS Curriculum for Mental Health in Education*. The evaluation of the first 2022/2023 cohort was highly satisfactory, and similarly positive outcomes are expected for the 2025/2026 academic year cohort. This program has proven to be a high-quality and timely response to the needs of contemporary students, as well as the needs of modern educators and teachers, particularly regarding their well-being and mental health within the educational context. Upon completing the program, participants acquire up-to-date competencies in mental health and psychological well-being, which they can effectively apply when working with children in early childhood, preschool, and primary school settings. Additionally, the program supports the strengthening of participants’ own mental health and well-being, which is crucial for the overall quality of the educational system in Croatia.

## Figures and Tables

**Table 1:** Socio-demographic variables of the subsamples.

		Phase	Frequency	Gender				School Type							
				M		F		K		PS		LSS		HSS	
Teachers’ ratings of students	Experimental	Pre	N = 404	202		202		179		121		46		58	
		Post	N = 404												
	Control	Pre	N = 386	207		179		150		93		95		48	
		Post	N = 386												
	Total		N = 790	409		381		329		214		141		106	
Teachers’ self-ratings	Experimental	Pre	N = 36	1		35		13		11		8		4	
		Post	N = 36												
	Control	Pre	N = 40	4		36		13		11		10		6	
		Post	N = 40												
	Total		N = 76	5		71		26		22		18		10	
Students’ self-ratings	Experimental							Primary school				Secondary school			
		Pre	N = 97	32		65		40				57			
		Post	N = 97												
	Control	Pre	N = 103	48		55		52				51			
		Post	N = 103												
	Total		N = 200	80		120		92				108			
				SES				Parents’ level of education							
Parents’ ratings of students	Experimental			L	M		H	P	S		PS		T		PG
		Pre	N = 211	83	71		57	10	94		26		67		14
		Post	N = 211												
	Control	Pre	N = 242	67	98		77	8	93		27		84		30
		Post	N = 242												
	Total		N = 453	150	169		134	18	187		53		151		44

Legend—Gender: M = male; F = female; School type: K = Kindergarten; PS = Primary school; LSS = Lowe secondary school; HSS = High secondary school; SES: L = Low; M = Medium; H = High; Parents’ level of education: P = Primary; S = Secondary; PS = Post secondary; T = Tertiary; and PG = Post graduate.

**Table 2:** Descriptive results (means and standard deviations) and results of testing the significance of differences (z-scores, *p*-values, and Cohen’s d) regarding the control (C) vs. experimental (E) group, and regarding phase 1 vs. phase 2 in each group on students’ internalized and externalized difficulties (1), prosocial behavior (2), self-awareness (3), self-management (4), social awareness (5), relationships (6), responsible decision making (7), and total SEC (8) based on teachers’ ratings.

	Phase 1		Phase 2		Control (N = 386)		Experimental (N = 404)	
	C	E	C	E	1	2	1	2
(1)	1.34 (0.29)	1.36 (0.29)	1.33 (0.29)	1.32 (0.29)	1.34 (0.29)	1.33 (0.29)	1.36 (0.29)	1.32 (0.29)
							Z = −3.008, *p* = 0.003, d = 0.292	
(2)	2.55 (0.45)	2.45 (0.47)	2.58 (0.44)	2.52 (0.48)	2.55 (0.45)	2.58 (0.44)	2.45 (0.47)	2.52 (0.48)
	Z = −3.092, *p* = 0.002, d = 0.462						Z = −2.420, *p* = 0.016, d = 0.474	
(3)	2.85 (0.56)	2.86 (0.54)	2.93 (0.53)	2.97 (0.59)	2.85 (0.56)	2.93 (0.53)	2.86 (0.54)	2.97 (0.59)
							Z = −2.891, *p* = 0.004, d = 0.563	
(4)	3.01 (0.58)	2.95 (0.65)	3.04 (0.55)	3.02 (0.66)	3.01 (0.58)	3.04 (0.55)	2.95 (0.65)	3.02 (0.66)
(5)	3.15 (0.63)	3.02 (0.60)	3.21 (0.59)	3.10 (0.64)	3.15 (0.63)	3.21 (0.59)	3.02 (0.60)	3.10 (0.64)
	Z = −3.163, *p* = 0.002, d = 0.613		Z = −2.142, *p* = 0.032, d = 0.615				Z = −2.110, *p* = 0.035, d = 0.620	
(6)	3.17 (0.58)	3.10 (0.59)	3.24 (0.56)	3.19 (0.59)	3.17 (0.58)	3.24 (0.56)	3.10 (0.59)	3.19 (0.59)
							Z = −2.059, *p* = 0.039, d = 0.591	
(7)	3.23 (0.61)	3.16 (0.61)	3.26 (0.57)	3.27 (0.62)	3.23 (0.61)	3.26 (0.57)	3.16 (0.61)	3.27 (0.62)
							Z = −2.779, *p* = 0.005, d = 0.617	
(8)	3.08 (0.52)	3.02 (0.50)	3.14 (0.49)	3.11 (0.54)	3.08 (0.52)	3.14 (0.49)	3.02 (0.50)	3.11 (0.54)
							Z = −2.570, *p* = 0.010, d = 0.525	

**Table 3:** Descriptive results (means and standard deviations) and results of testing the significance of differences (z-scores, *p*-values, and Cohen’s d) regarding the control (C) vs. experimental (E) group, and regarding phase 1 vs. phase 2 in each group on students’ internalized and externalized difficulties (1), prosocial behavior (2), self-awareness (3), self-management (4), social awareness (5), relationships (6), responsible decision making (7), and total SEC (8) based on parents’ ratings.

	Phase 1		Phase 2		Control (N = 242)		Experimental (N = 211)	
	C	E	C	E	1	2	1	2
(1)	1.35 (0.22)	1.32 (0.20)	1.35 (0.24)	1.27 (0.19)	1.35 (0.22)	1.35 (0.24)	1.32 (0.20)	1.27 (0.19)
			Z = −3.723, *p* = 0.000, d = 0.216				Z = −2.922, *p* = 0.003, d = 0.194	
(2)	2.71 (0.31)	2.71 (0.30)	2.70 (0.30)	2.75 (0.30)	2.71 (0.31)	2.70 (0.30)	2.71 (0.30)	2.75 (0.30)
(3)	3.00 (0.45)	3.07 (0.45)	3.07 (0.46)	3.15 (0.42)	3.00 (0.45)	3.07 (0.46)	3.07 (0.45)	3.15 (0.42)
							Z = −1.946, *p* = 0.05, d = 0.435	
(4)	2.74 (0.49)	2.81 (0.48)	2.83 (0.51)	2.88 (0.47)	2.74 (0.49)	2.83 (0.51)	2.81 (0.48)	2.88 (0.47)
					Z = −2.272, *p* = 0.023, d = 0.502			
(5)	3.25 (0.49)	3.28 (0.50)	3.28 (0.50)	3.33 (0.49)	3.25 (0.49)	3.28 (0.50)	3.28 (0.50)	3.33 (0.49)
(6)	3.28 (0.50)	3.35 (0.48)	3.34 (0.52)	3.42 (0.45)	3.28 (0.50)	3.34 (0.52)	3.35 (0.48)	3.42 (0.45)
(7)	3.26 (0.51)	3.29 (0.51)	3.28 (0.48)	3.38 (0.47)	3.26 (0.51)	3.28 (0.48)	3.29 (0.51)	3.38 (0.47)
			Z = −2.121, *p* = 0.034, d = 0.477					
(8)	3.11 (0.38)	3.16 (0.38)	3.16 (0.37)	3.23 (0.35)	3.11 (0.38)	3.16 (0.37)	3.16 (0.38)	3.23 (0.35)
			Z = −2.025, *p* = 0.043, d = 0.361				Z = −2.114, *p* = 0.035, d = 0.365	

**Table 4:** Descriptive results (means and standard deviations) and results of testing the significance of differences (z-scores, *p*-values, and Cohen’s d) regarding the control (C) vs. experimental (E) group, and regarding phase 1 vs. phase 2 in each group on students’ internalized and externalized difficulties (1), prosocial behavior (2), self-awareness (3), self-management (4), social awareness (5), relationships (6), responsible decision making (7), total SEC (8), and resilience (9) based on their self-ratings.

	Phase 1		Phase 2		Control (N = 103)		Experimental (N = 97)	
	C	E	C	E	1	2	1	E
(1)	1.54 (0.25)	1.53 (0.27)	1.55 (0.26)	1.53 (0.28)	1.54 (0.25)	1.55 (0.26)	1.53 (0.27)	1.53 (0.28)
(2)	2.56 (0.38)	2.71 (0.35)	2.60 (0.35)	2.68 (0.33)	2.56 (0.38)	2.60 (0.35)	2.71 (0.35)	2.68 (0.33)
	Z = −2.161, *p* = 0.031, d = 0.362							
(3)	3.17 (0.45)	3.20 (0.50)	3.29 (0.44)	3.32 (0.40)	3.17 (0.45)	3.29 (0.44)	3.20 (0.50)	3.32 (0.40)
					Z = −2.001, *p* = 0.045, d = 0.443			
(4)	3.02 (0.47)	2.97 (0.47)	3.05 (0.48)	3.10 (0.43)	3.02 (0.47)	3.05 (0.48)	2.97 (0.47)	3.10 (0.43)
(5)	3.33 (0.53)	3.39 (0.52)	3.35 (0.50)	3.45 (0.50)	3.33 (0.53)	3.35 (0.50)	3.39 (0.52)	3.45 (0.50)
(6)	3.41 (0.46)	3.43 (0.31)	3.41 (0.46)	3.44 (0.39)	3.41 (0.46)	3.41 (0.46)	3.43 (0.31)	3.44 (0.39)
(7)	3.39 (0.39)	3.33 (0.39)	3.45 (0.40)	3.44 (0.39)	3.39 (0.39)	3.45 (0.40)	3.33 (0.39)	3.44 (0.39)
							Z = −2.103, *p* = 0.035, d = 0.391	
(8)	3.26 (0.35)	3.26 (0.34)	3.31 (0.35)	3.35 (0.34)	3.26 (0.35)	3.31 (0.35)	3.26 (0.34)	3.35 (0.34)
							Z = −1.957, *p* = 0.05, d = 0.340	
(9)	3.83 (0.62)	3.76 (0.50)	3.77 (0.57)	3.83 (0.54)	3.83 (0.62)	3.77 (0.57)	3.76 (0.50)	3.83 (0.54)

**Table 5:** Descriptive results (means and standard deviations) and results of testing the significance of differences (z-scores, *p*-values, and Cohen’s d) regarding the control (C) vs. experimental (E) group, and regarding phase 1 vs. phase 2 in each group on teachers’ variables including social and emotional competencies (1), resilience (2), self-efficacy (student engagement (3), instructional strategies (4), and classroom management (5)), and burnout (6) based on their self-ratings.

	Phase 1		Phase 2		Control (N = 40)		Experimental (N = 36)	
	C	E	C	E	1	2	1	2
(1)	4.86 (0.39)	4.97 (0.32)	4.90 (0.33)	5.05 (0.37)	4.86 (0.39)	4.90 (0.33)	4.97 (0.32)	5.05 (0.37)
			Z = −2.207, *p* = 0.027, d = 0.348					
(2)	3.97 (0.48)	3.88 (0.44)	4.01 (0.42)	4.14 (0.43)	3.97 (0.48)	4.01 (0.42)	3.88 (0.44)	4.14 (0.43)
							Z = −2.447, *p* = 0.014, d = 0.433	
(3)	6.72 (1.03)	6.90 (0.97)	6.78 (0.89)	7.33 (0.76)	6.72 (1.03)	6.78 (0.89)	6.90 (0.97)	7.33 (0.76)
			Z = −2.553, *p* = 0.011, d = 0.834				Z = −2.041, *p* = 0.041, d = 0.872	
(4)	6.76 (0.97)	6.88 (0.99)	7.03 (0.79)	7.46 (0.64)	6.76 (0.97)	7.03 (0.79)	6.88 (0.99)	7.46 (0.64)
			Z = −2.436, *p* = 0.015, d = 0.721				Z = −2.425, *p* = 0.015, d = 0.835	
(5)	7.01 (0.83)	7.26 (0.92)	7.06 (0.74)	7.67 (0.69)	7.01 (0.83)	7.06 (0.74)	7.26 (0.92)	7.67 (0.69)
			Z = −3.410, *p* = 0.001, d = 0.714				Z = −2.032, *p* = 0.042, d = 0.814	
(6)	2.80 (0.91)	3.03 (1.06)	2.98 (1.07)	2.64 (0.68)	2.80 (0.91)	2.98 (1.07)	3.03 (1.06)	2.64 (0.68)

## Data Availability

The data underlying this article cannot be shared publicly because of the privacy and confidentiality of the study participants, which are protected. The data of this study are available upon reasonable request from the corresponding author.
